# Free fatty acids stabilize integrin β_1_*via* S-nitrosylation to promote monocyte–endothelial adhesion

**DOI:** 10.1016/j.jbc.2022.102765

**Published:** 2022-12-05

**Authors:** Qinyu Yao, Qi Cui, Jia Liu, Xinya Xie, Tingting Jiang, Haodong Wang, Ziwei Zhao, Wenfei Zhao, Xiong Du, Baochang Lai, Lei Xiao, Nanping Wang

**Affiliations:** 1 Cardiovascular Research Center, School of Basic Medical Sciences, Xi’an Jiaotong University, Xi’an, China; 2 Key Laboratory of Environment and Genes Related to Diseases, Xi'an Jiaotong University, Ministry of Education of China, Xi’an, China; 3 Advanced Institute for Medical Sciences, Dalian Medical University, Dalian, China; 4 East China Normal University, Health Science Center, Shanghai, China

**Keywords:** nitric oxide, S-nitrosylation, integrin, macrophage, endothelial cell, CHX, cycloheximide, FBS, fetal bovine serum, Fe(DETC)_2_, Fe^2+^ diethyldithiocarbamate, FFA, free fatty acid, GSNO, S-nitrosoglutathion, IBP, irreversible biotinylation procedure, iNOS, inducible nitric oxide synthase, NAC, N-Acetyl-L-cysteinamide, NO, nitric oxide, PTIO, 2-Phenyl-4,4,5,5-tetramethylimidazoline-1-oxyl 3-oxide, RT-qPCR, RNA extraction and reverse transcriptase-PCR, SNP, sodium nitroprusside

## Abstract

Hyperlipidemia characterized by high blood levels of free fatty acids (FFAs) is important for the progression of inflammatory cardiovascular diseases. Integrin β_1_ is a transmembrane receptor that drives various cellular functions, including differentiation, migration, and phagocytosis. However, the underlying mechanisms modifying integrin β_1_ protein and activity in mediating monocyte/macrophage adhesion to endothelium remain poorly understood. In this study, we demonstrated that integrin β_1_ protein underwent S-nitrosylation in response to nitrosative stress in macrophages. To examine the effect of elevated levels of FFA on the modulation of integrin β_1_ expression, we treated the macrophages with a combination of oleic acid and palmitic acid (2:1) and found that FFA activated inducible nitric oxide synthase/nitric oxide and increased the integrin β_1_ protein level without altering the mRNA level. FFA promoted integrin β_1_ S-nitrosylation *via* inducible nitric oxide synthase/nitric oxide and prevented its degradation by decreasing binding to E3 ubiquitin ligase c-Cbl. Furthermore, we found that increased integrin α_4_β_1_ heterodimerization resulted in monocyte/macrophage adhesion to endothelium. In conclusion, these results provided novel evidence that FFA-stimulated N--O stabilizes integrin β_1_*via* S-nitrosylation, favoring integrin α_4_β_1_ ligation to promote vascular inflammation.

Hyperlipidemia manifested by high blood levels of free fatty acids (FFAs) is critical for the progression of multiple metabolic diseases as well as cardiovascular complications. FFA levels increase significantly in obesity, type 2 diabetes, and atherosclerosis (1, 2, 3). Increased FFA level causes lipotoxicity and activates the release of proinflammatory cytokines such as IL-6, TNFα, and other endogenous endotoxin. These events are not only thought to link metabolic inflammation but also are even considered as cause of atherosclerosis and hypertension. In response to increased lipid levels, the endothelial cells release chemokines, whereas the monocyte/macrophages develop oxidative stress and upregulate chemokine receptor.

Inducible nitric oxide synthase (iNOS) is the type of NOS expressed in monocyte/macrophage and plays a critical role in physiopathological processes ranging from antimicrobial and wound healing to proinflammatory diseases. Expression of iNOS is negligible in resting cells and induced upon infection or metabolic stress, such as elevated levels of glucose and lipid (4). Accumulating evidence strongly implies that iNOS-derived nitric oxide (NO) plays a central role in the regulation of lipid metabolism during inflammatory conditions (5), resulting in endothelial dysfunction, atherosclerosis, or hypertension (6, 7, 8).

Adhesive interactions between immune and other cell types are largely mediated by integrins. Mammalian integrins comprise 18 α and eight β subunits that constitute 24 heterodimers, which have been implicated in either acute (9) or chronic metabolic inflammation (10). Integrin β_1_ serves a critical function in cell adhesion and occurs on numerous cells of the immune system (11). The evidence that integrin α_4_β_1_ heterodimer modulates metabolic inflammation in obesity implies its role in leukocyte recruitment (12). Integrin α_4_β_1_, presented on the leukocyte membrane, binds VCAM-1 on endothelial cells (13, 14). Rapid chemokine-increased integrin α_4_β_1_ affinity is required for monocyte arrest (15). During inflammation, integrin α_4_β_1_ drives transendothelial leukocyte migration into the inflamed tissue (16). In addition, integrin α_4_β_1_ can interact with vascular endothelial growth factor receptor 2, contributing to vascular endothelial growth factor functions (17). Integrin β_1_ expression is regulated during diverse pathophysiological processes including infection, tumorigenesis, and asthma (18, 19, 20). A change in the expression of integrin receptors usually takes place at the transcription level. However, integrin β_1_ protein undergoes posttranslational modifications, including phosphorylation (21), acetylation (22) and ubiquitination (23). Our understanding of the other posttranslational modification of integrin β_1_ is limited. Protein S-nitrosylation is a redox sensitive reaction at cysteine residues by NO or NO-derived species to generate a S-nitrosothiol (24). Previous reports suggest that certain posttranslational modification may regulate protein ubiquitination and function. S-nitrosylation may promote or inhibit degradation of certain protein such as dihydrofolate reductase (25) or TRIM72 (26), respectively. S-nitrosylation of integrin α_6_ decreases its binding to laminin-β_1_ and increases the extent of prostate cancer cell migration (27). Thus, whether integrin β_1_ could be regulated through S-nitrosylation remains as a critical question to understand its signaling transduction and function.

In this study, we demonstrated that iNOS-generated NO stabilizes integrin β_1_ *via* S-nitrosylation, favorizing integrin α_4_β_1_ ligation and monocyte/macrophage adhesion to endothelium.

## Results

### FFA promotes iNOS-dependent NO release in macrophages

**Figure 1:**
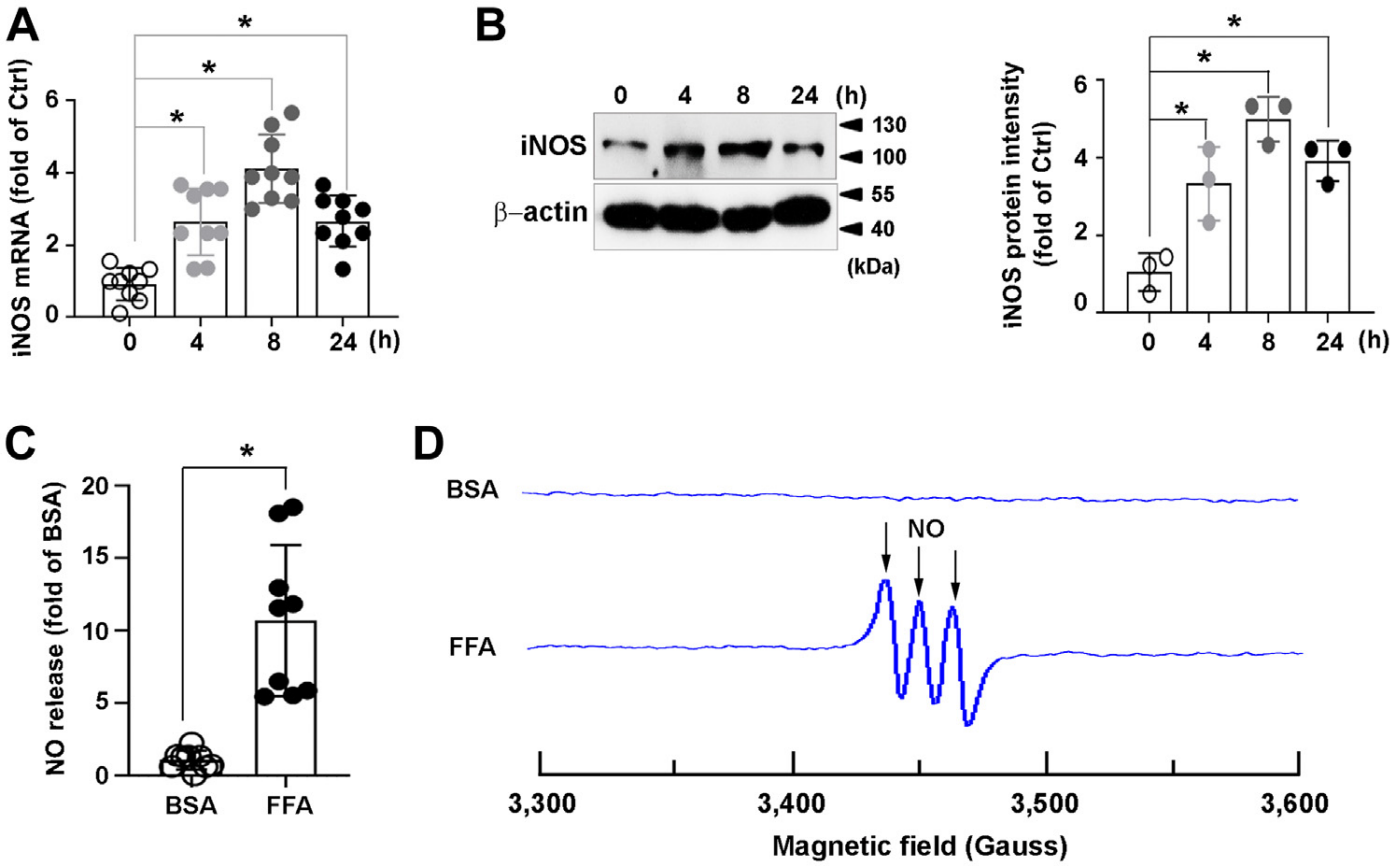
**Expression and activity of iNOS in FFA-affected macrophages.** Raw264.7 cells were treated with FFA (1 mM) for indicated times. *A* and *B*, cell lysates were analyzed for the levels of iNOS using (*A*) RT-qPCR and (*B*) immunoblotting (*left*); *C*, NO release in the medium of FFA-treated Raw264.7 for 24 h was measured by using Griess reaction; *D*, NO production was measured with EPR using Fe(DETC)_2_. Data of RT-qPCR or NO quantification by Griess reaction were from three independent experiments performed in triplicate. Immunoblots shown were representative of three independent experiments. Quantifications of band intensity were normalized to β-actin (*right*). Spectra shown were representative for three separate experiments. ∗*p* < 0.05. EPR, electron paramagnetic resonance; Fe(DETC)_2,_ Fe^2+^ diethyldithiocarbamate; FFA, free fatty acid; iNOS, inducible nitric oxide synthase; NO, nitric oxide.

Expression of iNOS is upregulated upon metabolic perturbation and considered as a marker of the classical and proinflammatory activation of macrophages (4). To investigate whether high level of FFA could induce inflammation in macrophages, Raw264.7 cells were subjected to FFA for the indicated times. FFA increased the levels of iNOS mRNA (Fig. 1*A*) and protein (Fig. 1*B*) 4 h after treatment. Then, the effect of FFA on NO production was examined in macrophages. As shown in Fig. 1*C*, treatment with FFA significantly augmented NO quantity in the conditioned media of macrophages. This result was further verified using an electron paramagnetic resonance (EPR) spectroscopy (Fig. 1*D*). These data demonstrated that iNOS expression was upregulated by FFA in macrophages.

### iNOS silencing reduces integrin β_1_ protein level in macrophages

**Figure 2:**
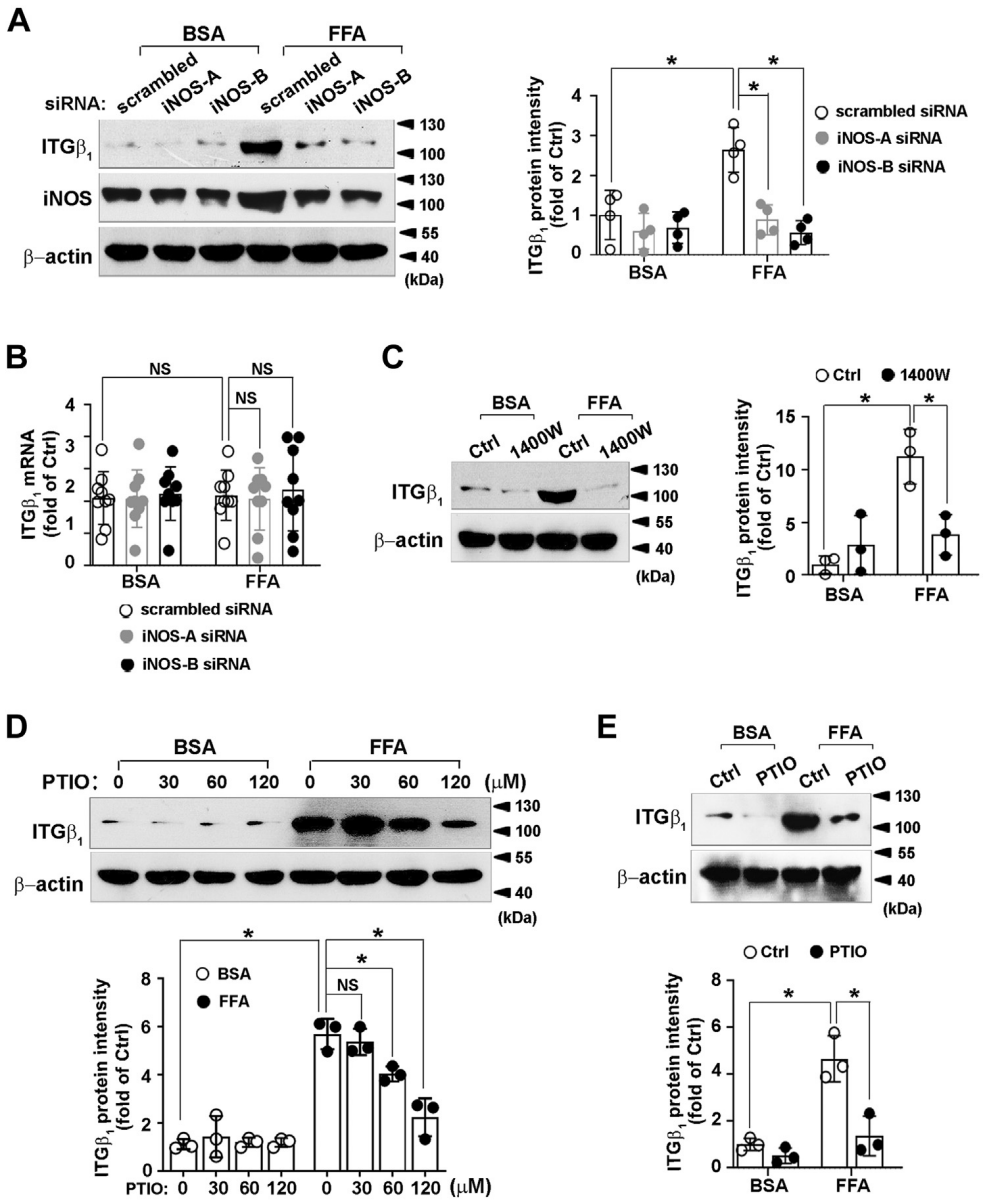
**Effect of iNOS/NO axis on integrin β**_**1**_**modulation.** Raw264.7 cells were transfected with scrambled siRNA or two pairs of iNOS siRNA (iNOS-A and iNOS-B), then incubated with FFA (1 mM) for 24 h. *A* and *B*, the cell lysates were analyzed for the protein level of iNOS, integrin β_1_ and β-actin by using immunoblotting (*A*, *left*) or for the level of integrin β_1_ mRNA by RT-qPCR (*B*); *C* and *D*, Raw264.7 cells were pretreated with 1400W (*C*, *left*) or with the indicated concentrations of PTIO (*D*, *up*) for 1 h, then exposed to FFA (1 mM) for 24 h; *E*, cells were incubated with FFA (1 mM) for 24 h after pretreatment with PTIO (60 μM) for 1 h (*up*). The cell lysates were analyzed with integrin β_1_ and β-actin antibodies by using immunoblotting *(A* and *C*: *right*; *D* and *E*: *down*). Immunoblots shown were representative of three independent experiments. Quantifications of band intensity were normalized to β-actin *(A* and *C*: *right*; *D* and *E*: *down*). ∗*p* < 0.05. FFA, free fatty acid; iNOS, inducible nitric oxide synthase; ITGβ_1_, integrin β_1_; NO, nitric oxide; NS, not significant; PTIO, 2-Phenyl-4,4,5,5-tetramethylimidazoline-1-oxyl 3-oxide.

iNOS plays a critical role in inflammatory progression, promoting us to evaluate its precise regulation on integrin β_1_. Raw264.7 cells were transfected with scrambled siRNA or two pairs of iNOS-specific siRNA. Both iNOS siRNAs markedly reduced iNOS protein compared to those transfected with scrambled siRNA, indicating their specificity. iNOS silencing reduced FFA-increased integrin β_1_ protein level compared to that of macrophages transfected with scrambled siRNA (Fig. 2*A*). However, the integrin β_1_ mRNA level was not altered (Fig. 2*B*). Similar to the results of iNOS siRNA silencing, 1400W, a selective iNOS inhibitor, ablated FFA-induced integrin β_1_ expression (Fig. 2*C*). These data suggested that iNOS participated in FFA-increased integrin β_1_ protein level in macrophages.

### iNOS-derived NO prevents integrin β_1_ reduction in macrophages

Next, we investigated the effect of iNOS-derived NO on integrin β_1_ expression. Raw264.7 cells were exposed to 2-Phenyl-4,4,5,5-tetramethylimidazoline-1-oxyl 3-oxide (PTIO), a known NO scavenger. Treatment of macrophages with PTIO (60 μM and 120 μM) for 24 h abolished FFA-induced integrin β_1_ protein level (Fig. 2*D*) without affecting iNOS expression (Fig. S1). Exposure of macrophages to PTIO (60 μM) for 24 h significantly reduced FFA-induced integrin β_1_ (Fig. 2*E*). These data suggested that FFA-induced NO augmented integrin β_1_ protein level.

### Integrin β_1_ is modulated by S-nitrosylation

**Figure 3:**
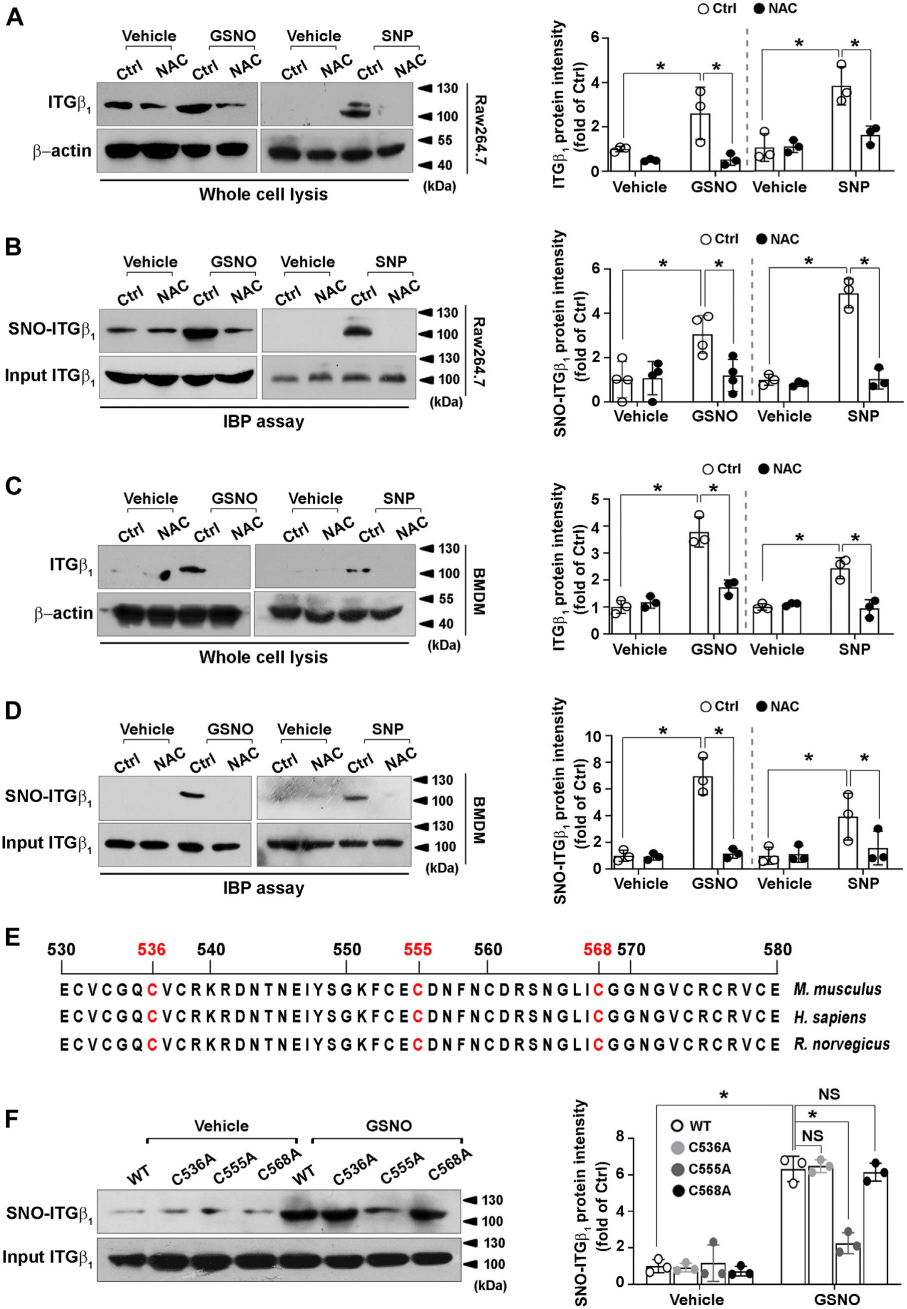
**S-nitrosylation of integrin β**_**1**_**.** Raw264.7 cells (*A*) or murine BMDMs (*C*) were pretreated with or without NAC (10 mM) for 2 h before exposure to GSNO (1 mM) or SNP (500 μM) for 24 h. Immunoblotting was performed to detect integrin β_1_ and β-actin (*left*); Before incubation with GSNO (1 mM, 4 h) or SNP (500 μM, 30 min) to trigger S-nitrosylation, Raw264.7 cells (*B*) *or murine BMDMs* (*D*) were pretreated with NAC (10 mM) for 2 h. IBP assay was performed, followed by immunoblotting to detect S-nitrosylated and total integrin β_1_ (*left*); *E*, amino acid sequences comparison of the cysteine-rich tandem domain of integrin β_1_ among species; *F*, HEK 293 cells were transfected with the wildtype (WT) or integrin β_1_ mutants (C536A, C555A, or C568A) then treated with GSNO (1 mM) for 4 h. S-nitrosylated integrin β_1_ was analyzed by IBP (*left*). Quantifications of band intensity normalized to β-actin *(A* and *C*: *right*) *or* total integrin β_1_ (*B*, *D* and *F*: *right*). Immunoblots shown were representative of three independent experiments. ∗*p* < 0.05. BMDM, bone marrow–derived macrophages; GSNO, S-nitrosoglutathion; IBP, irreversible biotinylation procedure; ITGβ, integrin β1; NAC, N-acetyl-L-cysteinamide; NS, not significant; SNP, sodium nitroprusside.

Since both iNOS silencing and NO scavenging lowered integrin β_1_ protein without altering its transcription, we reasoned whether NO would modulate integrin β_1_ protein. As depicted in Fig. 3*A*, exposure of Raw264.7 cells to S-nitrosoglutathion (GSNO) or organic salt sodium nitroprusside (SNP), two NO donors, augmented integrin β_1_ protein level, which was abolished by a reductant N-acetyl-L-cysteinamide (NAC). Taken together, these results suggested that NO might modulate integrin β_1_ protein level *via* a redox-sensitive reaction, promoting us to examine whether integrin β_1_ could be modified by S- nitrosylation. Raw264.7 cells were exposed to GSNO or SNP for 4 h and then subjected to irreversible biotinylation procedure. Immunoblotting showed marked S-nitrosylation of integrin β_1_, which was abrogated by NAC treatment (Fig. 3*B*). These data suggested that integrin β_1_ could be S-nitrosylated. Experiments conducted in murine bone marrow–derived macrophages also confirmed that NO might modulate integrin β_1_ protein level (Fig. 3*C*) *via* a S-nitrosylation-dependent mechanism (Fig. 3*D*).

Integrin β_1_ protein contains cysteine-rich tandem repeats which are crucial for its activation (28). As shown in Figure 3*E*, three cysteine residues (Cys536, Cys555, and Cys568), highly conserved among species, were predicted as potential S-nitrosylated sites by using three different web-based tools (iSNO-PseACC, iSNO-AAPair, and PreSNO). To further confirm the function residue that undergo S-nitrosylation, we replaced the three corresponding cysteines in integrin β_1_ with alanine (C536A, C555A, and C568A) by using site-directed mutagenesis. The expression plasmids of the wildtype or mutated integrin β_1_ was transfected into HEK 293 cells and then treated with GSNO. We found that integrin β_1_ mutation at C555A, but not C536A or C568A abolished GNSO-induced S-nitrosylation (Fig. 3*F*), suggesting that S-nitrosylation modified integrin β_1_ predominantly at Cys555.

### NO prevents its degradation *via* a ubiquitin-dependent mechanism

**Figure 4:**
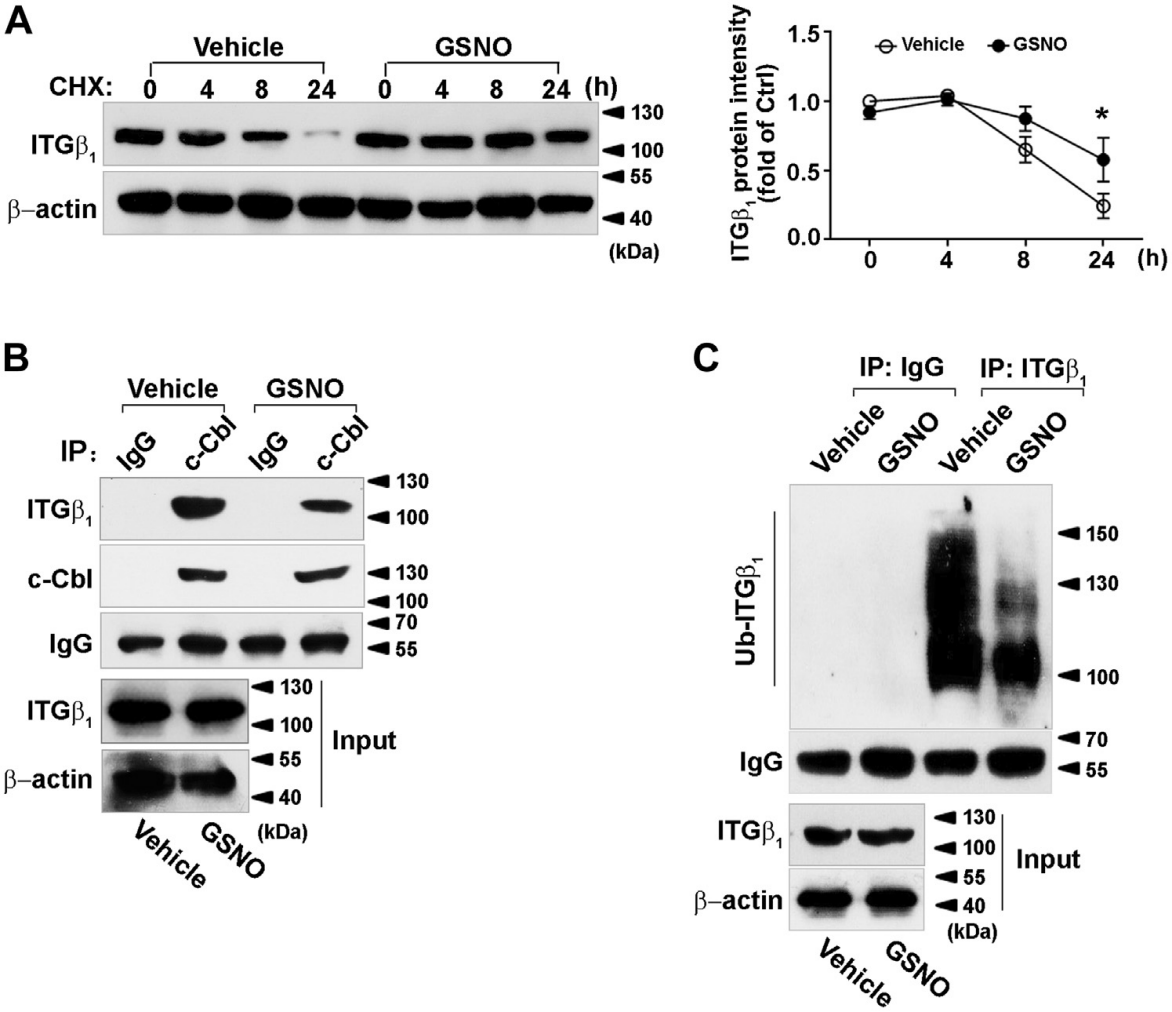
**Effect of S-nitrosylation on integrin β**_**1**_**ubiquitination and stability.** HEK 293 cells were transfected with integrin β_1_ overexpression plasmid. *A*, cells were pretreated with CHX (10 μg/ml) for 30 min before exposure to GSNO (1 mM) for indicated times, followed by immunoblotting to detect integrin β_1_ and β-actin (*left*). Quantifications of band intensity normalized to β-actin (*right*); *B*, immunoblotting of whole cell lysates (input) and immunoprecipitates (IP: c-Cbl) from cells with or without GSNO (1 mM) treatment for 24 h. MG132 (10 μM) was added to the medium 16 h before collecting; *C*, cells were pretreated with MG132 (10 μM) and then incubated with or without GSNO (1 mM) for 24 h. Integrin β_1_-was immunoprecipitated from cell lysates and immunoblotted with an anti-ubiquitin antibody. Immunoblots shown were representative of three independent experiments. ∗*p* < 0.05. CHX, cycloheximide; GSNO, S-nitrosoglutathion; ITGβ, integrin β1.

Because iNOS inhibitor attenuated the FFA-stimulated increase in integrin β_1_ protein level without affecting its mRNA level (Fig. 2*B*), we studied the effect of NO on integrin β_1_ protein stability. HEK 293 cells were transfected with plasmid expressing integrin β_1_. Then the cells were exposed to GSNO after pretreated with cycloheximide (CHX), an inhibitor of *de novo* protein synthesis. As shown in Figure 4*A*, GSNO increased the half-life of integrin β_1_ protein.

It is reported that integrin β_1_ degradation was mediated by E3 ubiquitin ligase c-Cbl (23). Co-immunoprecipitation indicated that the binding of c-Cbl to integrin β_1_ was largely diminished by GSNO treatment (Fig. 4*B*), which is consistent with the result of ubiquitination. As depicted in Figure 4*C*, GSNO decreased the detection of ubiquitinated integrin β_1_, suggesting that NO maintained integrin β_1_ stability by decreasing c-Cbl-dependent ubiquitination.

### FFA upregulates integrin β_1_ protein level *via* S-nitrosylation

**Figure 5:**
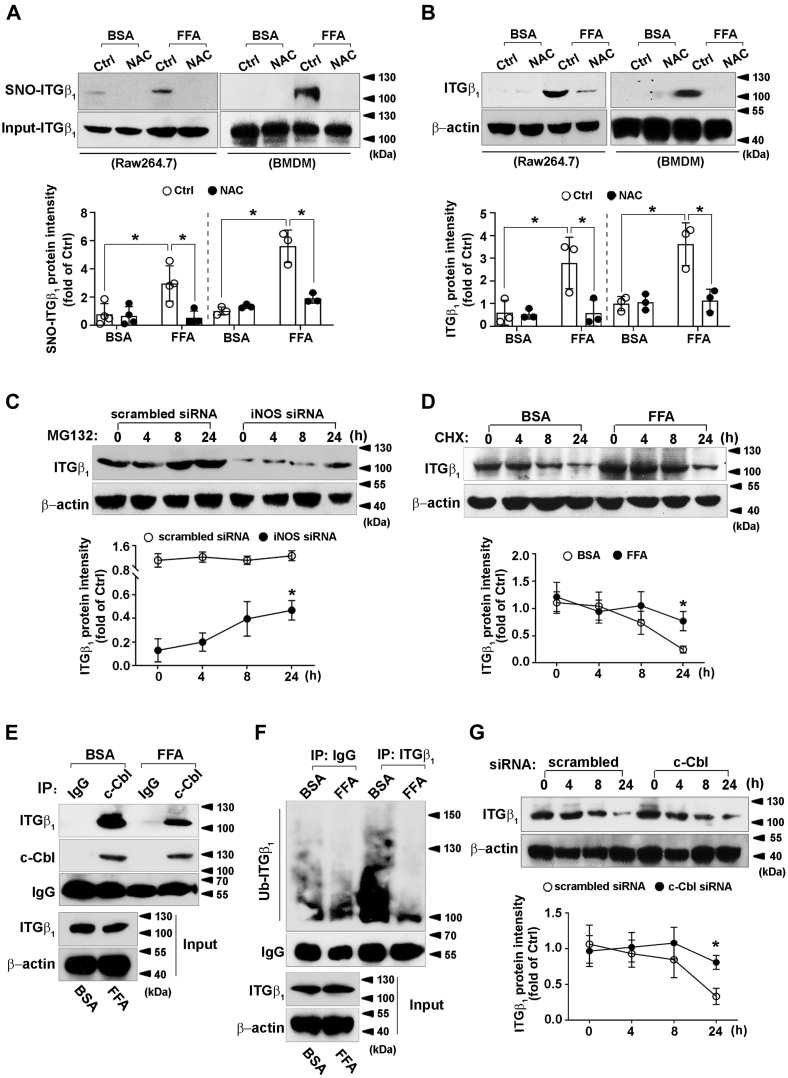
**Effect and mechanism of FFA on integrin β**_**1**_**modulation.***A*, Raw264.7 cells or murine BMDMs were pretreated with NAC (10 mM) for 2 h, followed by FFA incubation (Raw264.7: 4 h and BMDM: 12 h) to promote S-nitrosylation. IBP assay was then executed. The S-nitrosylated and total integrin β_1_ was detected by immunoblotting (*up*); *B*, Raw264.7 cells or murine BMDMs were pretreated with or without NAC (10 mM) for 2 h and then exposed to FFA (1 mM) for 24 h, followed by immunoblotting to detect integrin β_1_ and β-actin (*up*); *C*, scrambled or iNOS (pair B)-transfected Raw264.7 cells were incubated with FFA (1 mM) for 24 h, followed by MG132 (10 μM) treatment for indicated times (*up*); *D*, Raw264.7 cells were pretreated with CHX (10 μg/ml) for 30 min before exposure to FFA (1 mM) for indicated times, followed by immunoblotting to detect integrin β_1_ and β-actin (*up*); *E*, immunoblotting (IB; integrin β_1_) of whole cell lysates (input) and immunoprecipitates (IP: c-Cbl) from Raw264.7 cells with or without FFA (1 mM) treatment for 24 h. MG132 (10 μM) was added to the medium 16 h before collecting; *F*, Raw264.7 cells were pretreated with MG132 (10 μM) and then incubated with or without FFA (1 mM) for 24 h. Integrin β_1_-was immunoprecipitated from cell lysates and immunoblotted with an anti-ubiquitin antibody. Immunoblots shown were representative of three independent experiments; *G*, scrambled or c-Cbl-transfected Raw264.7 cells were primarily treated with CHX (10 μg/ml) for 30 min before incubation with FFA (1 mM) for indicated times (*up*). Quantifications of band intensity normalized to total integrin β_1_ (*A*: *down*) or β-actin (*B–D*, and *G*: *down*). ∗*p* < 0.05. BMDM, bone marrow–derived macrophages; CHX, cycloheximide; GSNO, S-nitrosoglutathion; IBP, irreversible biotinylation procedure; NAC, N-acetyl-L-cysteinamide.

As increasement of integrin β_1_ protein level was dependent on iNOS-derived NO, we reasoned whether integrin β_1_ would be modified by S-nitrosylation. For this purpose, Raw264.7 and bone marrow–derived macrophages were exposed to FFA. Immunoblotting showed significant S-nitrosylation of integrin β_1_, which was abolished by NAC pretreatment (Fig. 5*A*). Similar with the result of GSNO, macrophages with FFA treatment after 24 h showed an increasement of integrin β_1_ protein level, which was ablated by NAC (Fig. 5*B*). These data suggested FFA could increase integrin β_1_ protein level *via* S-nitrosylation-dependent mechanism.

### FFA stabilizes integrin β_1_ protein *via* iNOS/NO-inhibited proteasome degradation

We next examined whether iNOS silencing leads to integrin β_1_ reduction *via* ubiquitin-proteasome degradation. As shown in Figure 5*C*, MG132 ablated integrin β_1_ reduction caused by iNOS silencing. Similar with the results of GSNO, FFA treatment significantly inhibited integrin β_1_ degradation in the presence of CHX (Fig. 5*D*). Co-immunoprecipitation indicated that the binding of E3 ubiquitin ligase c-Cbl to integrin β_1_ was significantly decreased by FFA treatment (Fig. 5*E*), which was consistent that FFA decreased the ubiquitination level of integrin β_1_ (Fig. 5*F*). To confirm the effect of c-Cbl on integrin β_1_ degradation, Raw264.7 cells were primarily transfected with scrambled or c-Cbl siRNA, followed with FFA treatment. As depicted in Figure 5*G*, c-Cbl silencing further stabilized integrin β_1_ protein in FFA-treated macrophages. These data indicated that FFA augmented integrin β_1_ stability by reducing c-Cbl mediated ubiquitination.

### FFA enhances integrin α_4_β_1_ ligation

**Figure 6:**
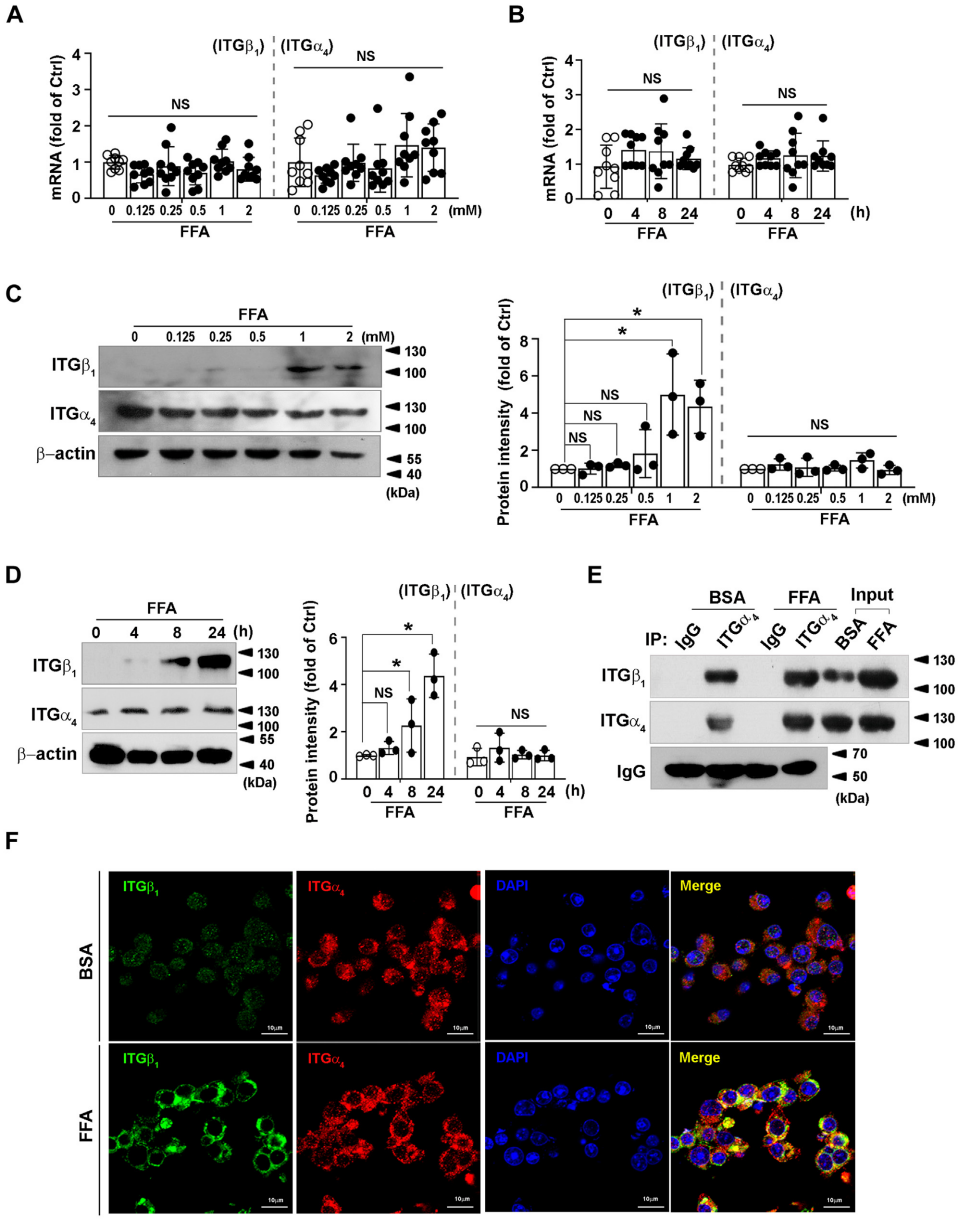
**Effect of FFA on integrin α**_**4**_**β**_**1**_**dimerization.***A–D*, Raw264.7 cells were stimulated with indicated concentrations of FFA for 24 h (*A* and *C*) or with FFA (1 mM) for the indicated times (*B* and *D*). The cell lysates were analyzed for the level of integrin β_1_ and α_4_ by using RT-qPCR or immunoblotting (*left*). *E*, immunoblotting (IB; integrin β_1_) of whole cell lysates (input) and immunoprecipitates (IP: integrin α_4_) from Raw264.7 cells with or without FFA (1 mM) treatment for 24 h. MG132 (10 μM) was added to the medium 16 h before collecting. *F*, *integrin* β_1_ (*green*) and α_4_ (*red*) in FFA-treated Raw264.7 cells were detected by immunostaining and imaged with confocal fluorescence microscope. Nuclei were counterstained using DAPI (*blue*). RT-qPCR data shown are from three independent experiments performed in triplicate. Immunoblots shown were representative of three independent experiments. Quantifications of band intensity were normalized to β-actin (*right*). ∗*p* < 0.05. FFA, free fatty acid; ITGβ, integrin β1; NS, not significant.

Given that integrins are obligate heterodimers and integrin α_4_ binds to integrin β_1_ in monocyte/macrophages (29), we examined the modulation of integrin α_4_ and β_1_ by FFA. FFA did not alter their mRNA expression (Fig. 6, *A* and *B*). Protein level of integrin β_1_ was induced by FFA in a concentration- (Fig. 6*C*) and time-dependent manner (Fig. 6*D*). In contrast, the protein level of integrin α_4_ remained unchanged (Fig. 6, *C* and *D*). Immunoprecipitation analysis was performed to examine the effect of FFA on integrin a_4_β_1_ dimerization. Their ligation was significantly enhanced in macrophages by FFA (Fig. 6*E*). Immunofluorescence staining on Raw274.7 cells confirmed that FFA upregulated integrin β_1_ protein level. Integrin α_4_ and β_1_ were localized on the surface of FFA-treated cells (Fig. 6*F*), suggesting that the formation of integrin α_4_β_1_ heterodimer on cellular membrane was increased by FFA in macrophages.

### FFA mediates adhesion of monocyte/macrophage to endothelium

**Figure 7:**
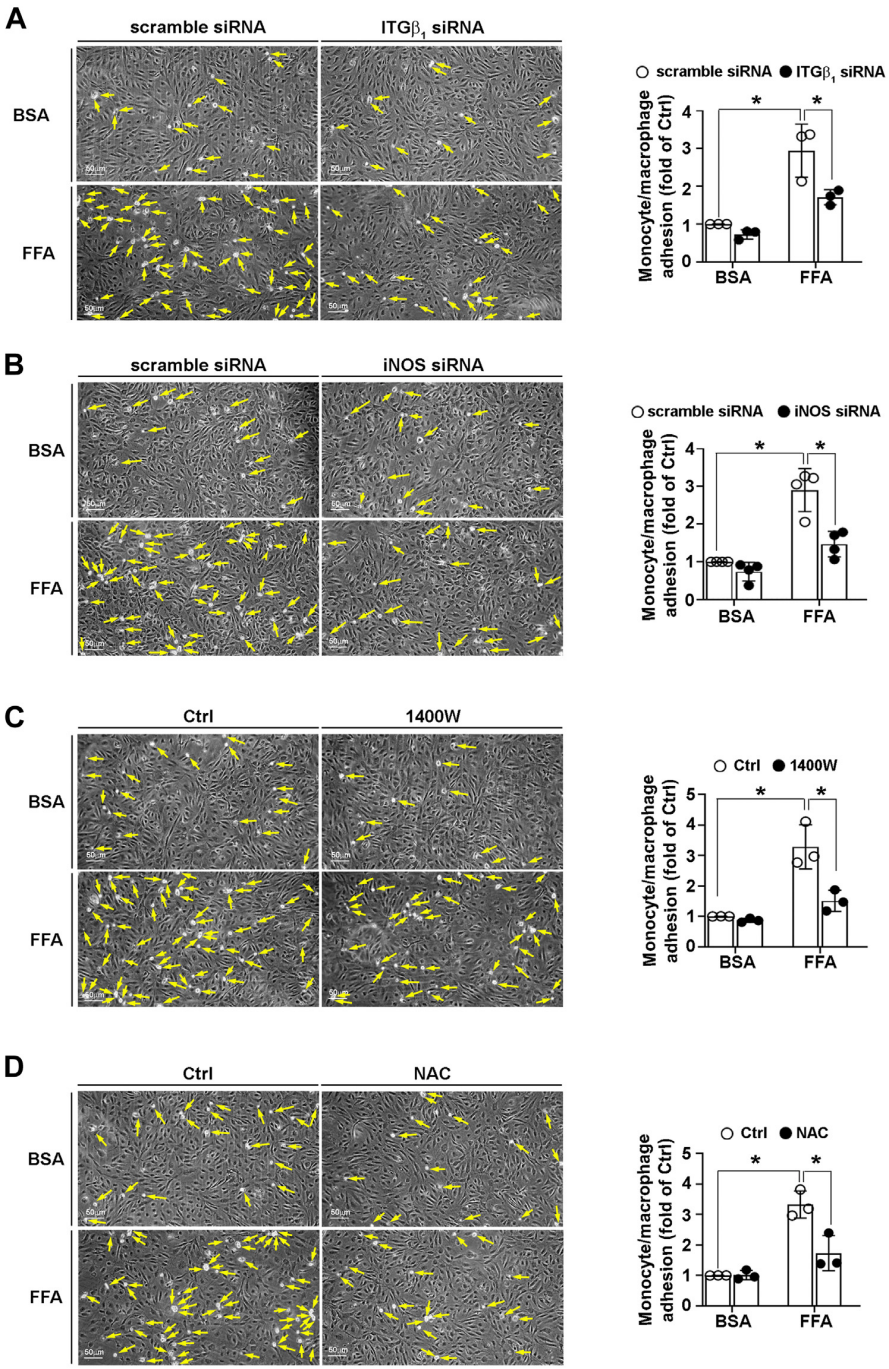
**Effect of integrin β**_**1**_**S-nitrosylation on monocyte/macrophage adhesion to endothelium.***A–D*, iNOS (*A*) or integrin β_1_ (*B*) siRNA-transfected, or 1400W (*C*), or NAC (*D*)-pretreated Raw264.7 cells were incubated with FFA (1 mM) for 24 h, then seeded on confluent endothelial cells. The attached Raw264.7 cells were pictured after 1 h. ∗*p* < 0.05. FFA, free fatty acid; iNOS, inducible nitric oxide synthase; ITGβ, integrin β1; NAC, N-acetyl-L-cysteinamide.

To investigate the function and related mechanism of integrin β_1_ in macrophages, FFA-treated Raw264.7 cells were primarily transfected with integrin β_1_ or iNOS siRNA. Either integrin β_1_ (Fig. 7*A*) or iNOS (Fig. 7*B*) siRNA markedly reduced the number of adhering macrophages to endothelial cells compared to those transfected with scrambled siRNA. Similar to the effect of iNOS siRNA, selective iNOS inhibitor, 1400W, abolished the stimulatory effect of FFA on monocyte/macrophage-endothelial interaction (Fig. 7*C*). In parallel, NAC pretreatment decreased FFA-induced monocyte/macrophage adhesion (Fig. 7*D*). These results indicated that integrin β_1_ participated in FFA-induced monocyte/macrophage to endothelium *via* iNOS/NO-dependent S-nitrosylation.

## Discussion

In this study, we revealed a novel modification of integrin β_1_, which is a key integrin subunit with essential roles in leukocyte extravascular migration and inflammatory responses. Here we demonstrated for the first time that elevated FFA triggered S-nitrosylation of integrin β_1_ in monocytes/macrophages. The decreased ubiquitination and proteasome-dependent degradation of integrin β_1_ enhanced monocyte–endothelial adhesion. Furthermore, these results provide mechanistic evidence that S-nitrosylation regulated the formation of a specific integrin heterodimer to mediate monocyte/macrophage adhesion to vascular endothelium.

Integrins are essential for the survival and migratory capacity of inflammatory cells. Integrin β_1_ upregulation is a major mechanism for monocyte/macrophage adhesion, whereas its downregulation is crucial for cell migration and metastasis (30). Therefore, integrin β_1_ serves as a switch to control cellular motility and recruitment. Integrin β_1_^+^ macrophages are highly detectable in carotid plaques of the patients with hyperlipidemia or in atherosclerotic lesions of ApoE^−/−^ mice (31, 32). Oxysterol, a cholesterol oxidation product, causes strong expression of integrin β_1_ in human macrophage lineage *via* c-Src/PLC/PKC/ERK pathway (33). Emerging evidence suggest that iNOS plays a critical role in metainflammation by creating nitrosative stress (34). S-nitrosylation of integrin α_6_ at Cys86 decreases its binding to laminin-β_1_, leading to decreased cell adherence and increased motility in prostate cancer cells (27). Exogenous NO modulates integrin α_IIb_β_3_ activation *via* a S-nitrosylation-dependent mechanism (35). However, the mechanism by which integrin undergoing S-nitrosylation is largely unknown. Our current study provided a mechanistic explanation as to how integrin β_1_ promoted monocyte-endothelial adhesion *via* S-nitrosylation, ubiquitination, and degradation. This finding may shed new light on the mechanisms by which dyslipidemia-associated metabolic perturbation leads to meta-inflammation.

Several studies suggest that posttranslational modifications are required for integrin β_1_ signaling transduction. Glycosylation protein controls integrin β_1_ activity and endocytic trafficking (22). Phosphorylation of tyrosine 788 in integrin β_1_ promotes inside-out receptor activation (36). Here, we found that S-nitrosylation inhibited integrin β_1_ degradation and triggers monocyte/macrophage adhesion to endothelium. It was reported in a redox proteome research that the Cys568 might be another potential cysteine residue undergoing S-nitrosylation in N-acetyl-p-aminophenol treated-hepatocytes (37). Both Cys555 and Cys568 are conserved among mouse, human, and rat. They are contained within the cysteine-rich tandem repeats which form disulfide bridge. The disulfide bridge is required for recognition of α subunit and critical for the transition from its inactive to active form (28). This is consistent with our finding that S-nitrosylation of integrin β_1_ regulated its ligation with the α_4_ subunit.

S-nitrosylation is reported to regulate protein ubiquitination and stability. Our research showed that S-nitrosylation decreased the binding between integrin β_1_ and E3 ubiquitin ligase c-Cbl, thereby inhibiting its ubiquitination and degradation. The mechanism by which S-nitrosylation prevents the interaction between integrin β_1_ and c-Cbl is to be investigated. A conformational change of integrin β_1_ might prevent recognition and subsequent attachment of ubiquitin. Other E3 ligases, such as Fbx2, are also involved in integrin β_1_ ubiquitination and proteasome-dependent degradation (30). Integrin β_1_ has been shown to be ubiquitinated, internalized, and degraded by the lysosome, a typical degradation pathway for transmembrane proteins (38). However, we found in this study that iNOS-derived NO prevented integrin β_1_ from proteasome-dependent degradation. It is tempting to speculate that the S-nitrosylation may mediate an unusual process for substrate recognition by the proteasome.

Integrin are heterodimers of α and β subunits, which are strictly ligated upon extracellular stimuli and required for its downstream signaling activation. The mechanism of integrin dimerization has not been well-understood. C1q-containing immune complexes is required for integrin α_2_β_1_ ligation in mast cells during innate immunity (39). Peroxisome proliferator-activated receptor γ inhibits integrin α_M_β_2_ and favorizes integrin α_V_β_5_ ligation during macrophage M2 polarization (40). Here, we found a regulatory effect of FFA-induced S-nitrosylation on integrin α_4_β_1_ ligation. To the best of our knowledge, this is the first evidence that S-nitrosylation leads to distinct ligation of specific integrin subunits.

Hyperlipidemia has been shown to trigger endothelium inflammation, characterized by the secretion of pro-inflammatory chemokine and recruitment of inflammatory cells. Integrin β_1_ present on monocyte/macrophage membranes plays a key role in facilitating the interaction with the activated endothelium (41). Blockage of integrin β_1_ suppresses diet-induced obesity by slowing down monocyte infiltration as well as by reducing the inflammatory responses of local macrophages (42). Oxidative and nitrosative stress has been implicated in endothelial dysfunction in high fat diet–fed mice (24). Except for the current knowledge of endothelial damage, we demonstrated integrin β_1_ S-nitrosylation enhanced monocyte/macrophage-endothelium interaction. Nevertheless, functional roles of integrin β_1_ S-nitrosylation in pathophysiological warrant further investigation.

E-Selectin or ICAM/VCAM are cellular adhesion molecules, present on the membrane of endothelial cells, and mediate the adhesion of leukocyte to endothelium (13). In contrast to the other posttranscriptional modification, S-nitrosylation is not strictly dependent on enzymes for its formation and breakage. This would result in a broad reactivity affecting many cysteine residues with a low specificity. We demonstrated a regulatory effect of S-nitrosylation on the formation of integrin α_4_β_1_ heterodimer. In parallel, S-nitrosylation of endothelial VCAM needs to be further investigated. Moreover, after binding to VCAM on the surface of endothelial cells, integrin β_1_ undergoes caveolar endocytosis and then routes for degradation (43). However, the regulation of S-nitrosylation on integrin β_1_ endocytosis warrants further investigation.

**Figure 8:**
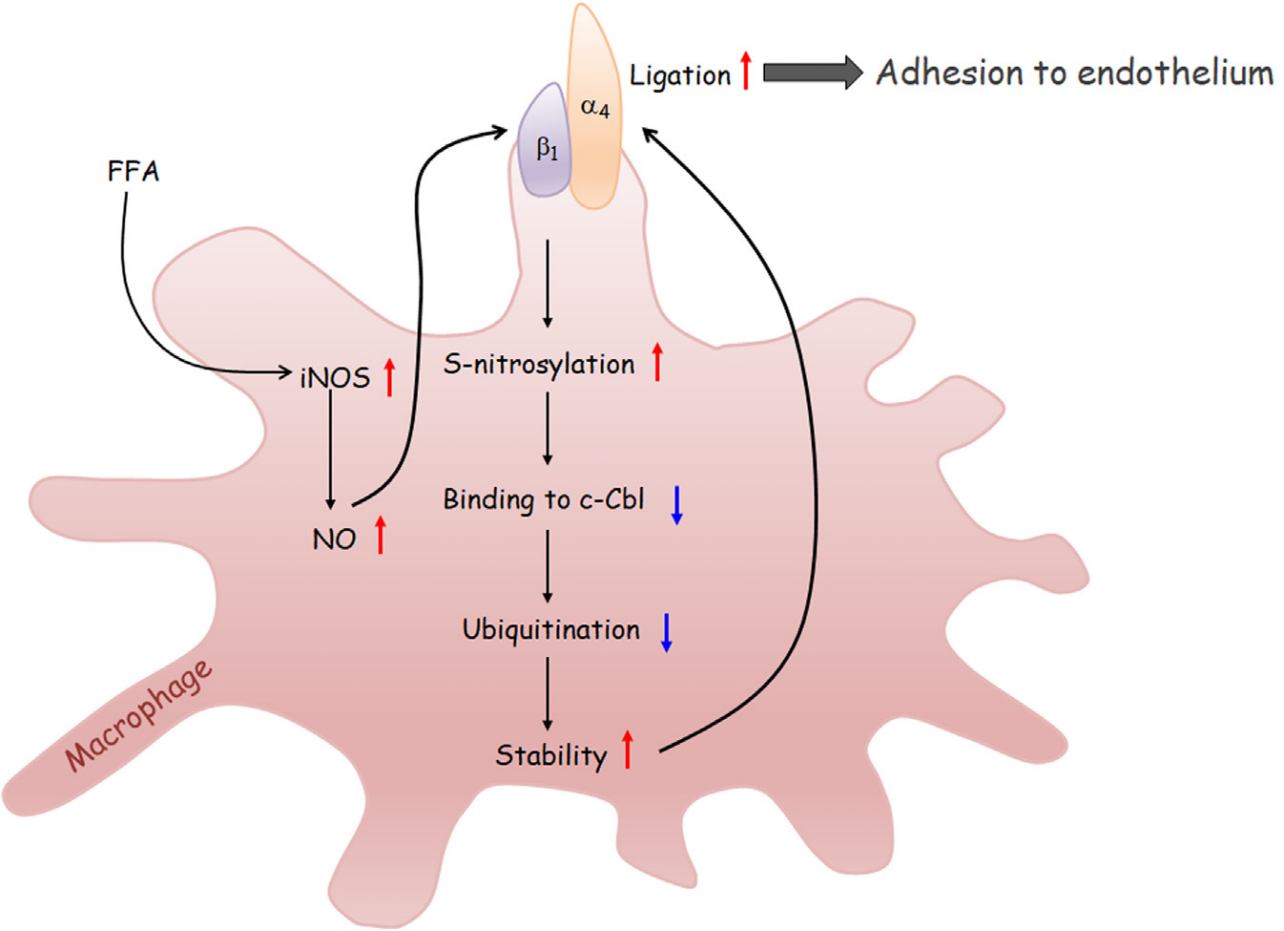
**Mechanism of integrin β**_**1**_**S-nitrosylation in monocyte/macrophage adhesion to endothelium.** FFA induces iNOS expression, resulting in increased NO release. The ensuing S-nitrosylation of integrin β_1_ deceases its binding to E3 ubiquitin ligase c-Cbl, leading to impaired ubiquitination and proteasome-dependent degradation. Enhanced integrin heterodimer α_4_β_1_ favorizes monocyte/macrophage adhesion to endothelium. FFA, free fatty acid; iNOS, inducible nitric oxide synthase; NO, nitric oxide.

Taken together, our data demonstrate that FFA-induced iNOS expression and NO release triggers S-nitrosylation, thus preventing integrin β_1_ degradation by decreasing its binding to E3 ubiquitin ligase c-Cbl. The ensuing ligation with integrin α_4_ leads to monocyte/macrophage adhesion to endothelium (Fig. 8), which may provide a potent target against inflammatory cardiovascular diseases.

## Experimental procedures

### Reagents

Primary antibodies against integrin β_1_ was from Cell signaling. Antibodies against integrin α_4_, iNOS, c-Cbl, ubiquitin, and β-actin were from Santa Cruz Biotechnology. 1400W, MG132, NAC, bovine serum albumin, PTIO, oleic acid, palmitic acid, and SNP were from Sigma-Aldrich. Granulocyte-macrophage colony-stimulating factor was from MedChemExpress. CHX was purchased from Selleck Chemicals. GSNO was synthesized from glutathione. To prepare the FFA mixture, oleic acid, and palmitic acid (2:1) were emulsified in PBS containing 1% bovine serum albumin.

### Cell culture

Murine monocytic cell line Raw264.7 and HEK 293 cells were obtained from ATCC and maintained in Dulbecco’s modified Eagle’s medium supplemented with 10% fetal bovine serum (FBS). Human umbilical vein endothelial cells were cultured in M199 medium with basic fibroblast growth factor and 20% FBS.

Bone marrow precursor cells were harvested from femurs of C57BL/6 mice, washed, and differentiated into macrophages in RPMI 1640 medium supplemented with 10% FBS and 20 ng/ml granulocyte-macrophage colony-stimulating factor. Cells were used 7 days after differentiation. The experiments were approved by the institutional ethics review board of Xi’an Jiaotong University (approval XJTULAC2018–497) and performed in accordance with the National Institutes of Health guidelines.

To detect macrophage-endothelial adhesion, Raw264.7 cells were seeded on confluent human umbilical vein endothelial cells and incubated for 1 h. After washing with PBS for 3 times, the attached Raw264.7 cells were pictured.

### Plasmids

Integrin β_1_ was subcloned into pcDNA3.1 plasmid. Mutagenesis of Cys536, Cys555, or Cys568 to alanine were performed from by using QuickChange@ Lightning Site-Directed Mutagenesis Kit (Agilent Technologies) and confirmed by sequencing. Plasmids were transfected using Lipofectamine 2000. Experiments using these cells were executed at 48 h after transfection.

### siRNA transfection

Raw264.7 cells were transfected with iNOS, integrin β_1_, c-Cbl, or scrambled siRNA. Sequences were described in Table S1. Experiments using these cells were executed at 24 h after transfection.

### RNA extraction and reverse transcriptase-PCR

Total RNA was extracted from Raw264.7 cells by using TRIzol (Invitrogen). Quantitative reverse transcriptase-PCR was performed using the SYBR Green technique (Promega). Primer sequences were described in Table S2. GAPDH was used as an internal control.

### Immunostaining

Cells were fixed in 4% paraformaldehyde. Detection of integrins was performed with anti-integrin β_1_ and α_4_ antibodies, then revealed with fluorescent dye (Alexa 488 and 555, respectively)–coupled secondary antibodies. Slides were mounted with DAPI-contained imaging medium. Images were captured with Nikon confocal microscope.

### Immunoblotting and immunoprecipitation

Proteins were extracted in RIPA buffer supplemented with protease inhibitors. Protein concentrations were measured using the BCA protein assay. Immunoblotting was performed with appropriate primary antibodies and horseradish peroxidase-conjugated secondary antibodies followed by ECL detection.

For immunoprecipitation, cell lysates were incubated with the appropriate antibodies or control IgG at 4 °C overnight followed by incubation with protein A/G-Sepharose beads. Immunoprecipitates were washed with NETN buffer (20 mM Tris, pH 8.0, 100 mM NaCl, 1 mM EDTA, and 0.5% NP-40). Immunoblotting was performed to detect by using corresponding antibodies.

### NO measurement by Griess reaction

The quantity of NO in the conditioned cell culture medium was evaluated by using Nitrate/Nitrie Colorimetric Assay Kit (Cayman Chemical). Briefly, the nitrate was conversed to nitrite utilizing nitrate reductase. Addition of the Griess Reagents converts nitrite into a deep purple azo compound. Photometric measurement of absorbance due to this azo chromophore determined NO_2_^−^ concentration, which was equal to the NO concentration.

### NO detection by EPR spectroscopy

NO detection was performed using the colloid spin-trap Fe^2+^ diethyldithiocarbamate [Fe(DETC)_2_] (200 μM) as previously described (44). FeSO4.7H_2_O was mixed with sodium DETC to generate Fe(DETC)_2_ colloid solution. Raw264.7 cells were incubated with [Fe(DETC)_2_] at 37 °C for 30 min. The NO- Fe(DETC)_2_ complex was detected with an EMX X-band spectrometer (Bruker Corporation) using the following parameters: microwave power, 10 mW; amplitude modulation, 7G; modulation frequency, 100 kHz; and three numbers of scans. The EPR signal was indicated as the amplitude of the triplet signal (45).

### S-nitrosylation detection by irreversible biotinylation procedure

The detection of integrin β_1_ S-nitrosylation in Raw264.7 cells was described as previously described (46). Briefly, protein was extracted in RIPA and precipitated in acetone and then subjected into HEN buffer (250 mM Hepes, 0.1 mM EDTA, 10 mM neocuproine) supplemented with 1% NP-40, protease inhibitors and 20 mM Methyl thiosulfonate. After centrifugation, free thiols were blocked by addition of 2.5% SDS. Proteins were reprecipitated in ice-cold acetone and dissolved in HEN buffer with 2.5% SDS, 0.2 mM biotin-maleimide, and 10 mM ascorbate to generation biotinylation. The biotinylated protein was precipitated in ice-cold acetone and resuspended in HEN buffer with 200 mM Dithiothreitol to destroy intermolecular disulfide bridges. The total biotinylated protein was precipitated with streptavidin-agarose and washed with HEN buffer containing 100 mM NaCl and 2.5% SDS. Samples were analyzed by immunoblotting to detect S-nitrosylated integrin β_1_. Band intensity was quantified and normalized to total integrin β_1_ (input). Immunoblots shown were representative of at least three independent experiments.

### Statistical analysis

The results are reported as means ± SD and analyzed using GraphPad Prism 8.0 software. The Student’s *t* test was used to determine the significant differences between two groups. Two-way analysis of variance (ANOVA), followed by Tukey post-hoc correction was used for the comparisons more than two groups. The protein stability test with CHX was analyzed by multiple-comparison two-way ANOVA followed by Bonferroni post-hoc correction.

## Data availability

All raw and processed data contained in the manuscript are available from the corresponding author upon request.

## Supporting information

This article contains supporting information.

## Conflict of interest

The authors declare no conflict of interest with the contents of the article.

## References

[1] Boden G. (2008). Obesity and free fatty acids. Endocrinol. Metab. Clin. North Am..

[2] Prescott J., Owens D., Collins P., Johnson A., Tomkin G.H. (1999). The fatty acid distribution in low density lipoprotein in diabetes. Biochim. Biophys. Acta.

[3] Rinne P., Guillamat-Prats R., Rami M., Bindila L., Ring L., Lyytikainen L.P. (2018). Palmitoylethanolamide promotes a proresolving macrophage phenotype and attenuates atherosclerotic plaque formation. Arterioscler Thromb. Vasc. Biol..

[4] Anavi S., Tirosh O. (2020). iNOS as a metabolic enzyme under stress conditions. Free Radic. Biol. Med..

[5] Rosas-Ballina M., Guan X.L., Schmidt A., Bumann D. (2020). Classical activation of macrophages leads to lipid droplet formation without de novo fatty acid synthesis. Front Immunol..

[6] Gliozzi M., Scicchitano M., Bosco F., Musolino V., Carresi C., Scarano F. (2019). Modulation of nitric oxide synthases by oxidized LDLs: role in vascular inflammation and atherosclerosis development. Int. J. Mol. Sci..

[7] Fysikopoulos A., Seimetz M., Hadzic S., Knoepp F., Wu C.Y., Malkmus K. (2021). Amelioration of elastase-induced lung emphysema and reversal of pulmonary hypertension by pharmacological iNOS inhibition in mice. Br. J. Pharmacol..

[8] Cromheeke K.M., Kockx M.M., De Meyer G.R., Bosmans J.M., Bult H., Beelaerts W.J. (1999). Inducible nitric oxide synthase colocalizes with signs of lipid oxidation/peroxidation in human atherosclerotic plaques. Cardiovasc. Res..

[9] Mezu-Ndubuisi O.J., Maheshwari A. (2021). The role of integrins in inflammation and angiogenesis. Pediatr. Res..

[10] Tian Y., Yang C., Yao Q., Qian L., Liu J., Xie X. (2019). Procyanidin B2 activates PPARgamma to induce M2 polarization in mouse macrophages. Front Immunol..

[11] Mrugacz M., Bryl A., Falkowski M., Zorena K. (2021). Integrins: an important link between angiogenesis, inflammation and eye diseases. Cells.

[12] Chung K.J., Chatzigeorgiou A., Economopoulou M., Garcia-Martin R., Alexaki V.I., Mitroulis I. (2017). A self-sustained loop of inflammation-driven inhibition of beige adipogenesis in obesity. Nat. Immunol..

[13] Gorina R., Lyck R., Vestweber D., Engelhardt B. (2014). Beta2 integrin-mediated crawling on endothelial ICAM-1 and ICAM-2 is a prerequisite for transcellular neutrophil diapedesis across the inflamed blood-brain barrier. J. Immunol..

[14] Chang A.C., Chen P.C., Lin Y.F., Su C.M., Liu J.F., Lin T.H. (2018). Osteoblast-secreted WISP-1 promotes adherence of prostate cancer cells to bone via the VCAM-1/integrin alpha4beta1 system. Cancer Lett..

[15] Hyduk S.J., Rullo J., Cano A.P., Xiao H., Chen M., Moser M. (2011). Talin-1 and kindlin-3 regulate alpha4beta1 integrin-mediated adhesion stabilization, but not G protein-coupled receptor-induced affinity upregulation. J. Immunol..

[16] Sackstein R. (2005). The lymphocyte homing receptors: gatekeepers of the multistep paradigm. Curr. Opin. Hematol..

[17] Gutierrez-Gonzalez A., Aguilera-Montilla N., Ugarte-Berzal E., Bailon E., Cerro-Pardo I., Sanchez-Maroto C. (2019). Alpha4beta1 integrin associates with VEGFR2 in CLL cells and contributes to VEGF binding and intracellular signaling. Blood Adv..

[18] Wang Y., Li K., Zhao W., Liu Z., Liu J., Shi A. (2021). Aldehyde dehydrogenase 3B2 promotes the proliferation and invasion of cholangiocarcinoma by increasing integrin beta 1 expression. Cell Death Dis.

[19] Teoh C.M., Tan S.S., Tran T. (2015). Integrins as therapeutic targets for respiratory diseases. Curr. Mol. Med..

[20] Speziale P., Pietrocola G. (2020). The multivalent role of fibronectin-binding proteins A and B (FnBPA and FnBPB) of staphylococcus aureus in host infections. Front Microbiol..

[21] Gahmberg C.G., Gronholm M. (2022). How integrin phosphorylations regulate cell adhesion and signaling. Trends Biochem. Sci..

[22] Mirgorodskaya E., Dransart E., Shafaq-Zadah M., Roderer D., Sihlbom C., Leffler H. (2022). Site-specific N-glycan profiles of alpha5 beta1 integrin from rat liver. Biol. Cell.

[23] Fan L., Zhang Y., Shi D., Xi R., Zhang Z., Wang X. (2021). Hypoxia enhances the cytotoxic effect of As4S4 on rat ventricular H9c2 cells through activation of ubiquitin-proteasome system. J. Trace Elem. Med. Biol..

[24] Chatterji A., Banerjee D., Billiar T.R., Sengupta R. (2021). Understanding the role of S-nitrosylation/nitrosative stress in inflammation and the role of cellular denitrosylases in inflammation modulation: implications in health and diseases. Free Radic. Biol. Med..

[25] Cai Z., Lu Q., Ding Y., Wang Q., Xiao L., Song P. (2015). Endothelial nitric oxide synthase-derived nitric oxide prevents dihydrofolate reductase degradation via promoting S-nitrosylation. Arterioscler Thromb. Vasc. Biol..

[26] Kohr M.J., Evangelista A.M., Ferlito M., Steenbergen C., Murphy E. (2014). S-nitrosylation of TRIM72 at cysteine 144 is critical for protection against oxidation-induced protein degradation and cell death. J. Mol. Cell Cardiol..

[27] Isaac J., Tarapore P., Zhang X., Lam Y.W., Ho S.M. (2012). Site-specific S-nitrosylation of integrin alpha6 increases the extent of prostate cancer cell migration by enhancing integrin beta1 association and weakening adherence to laminin-1. Biochemistry.

[28] Eble J.A., de Rezende F.F. (2014). Redox-relevant aspects of the extracellular matrix and its cellular contacts via integrins. Antioxid. Redox Signal.

[29] Hyduk S.J., Chan J.R., Duffy S.T., Chen M., Peterson M.D., Waddell T.K. (2007). Phospholipase C, calcium, and calmodulin are critical for alpha4beta1 integrin affinity up-regulation and monocyte arrest triggered by chemoattractants. Blood.

[30] Shi L., Liu B., Shen D.D., Yan P., Zhang Y., Tian Y. (2021). A tumor-suppressive circular RNA mediates uncanonical integrin degradation by the proteasome in liver cancer. Sci. Adv..

[31] Huang L.H., Melton E.M., Li H., Sohn P., Rogers M.A., Mulligan-Kehoe M.J. (2016). Myeloid Acyl-CoA:cholesterol Acyltransferase 1 deficiency reduces lesion macrophage content and suppresses atherosclerosis progression. J. Biol. Chem..

[32] Riek A.E., Oh J., Sprague J.E., Timpson A., de las Fuentes L., Bernal-Mizrachi L. (2012). Vitamin D suppression of endoplasmic reticulum stress promotes an antiatherogenic monocyte/macrophage phenotype in type 2 diabetic patients. J. Biol. Chem..

[33] Gargiulo S., Gamba P., Testa G., Sottero B., Maina M., Guina T. (2012). Molecular signaling involved in oxysterol-induced beta(1)-integrin over-expression in human macrophages. Int. J. Mol. Sci..

[34] Bruno A.S., Lopes P.D.D., de Oliveira K.C.M., de Oliveira A.K., de Assis Cau S.B. (2021). Vascular inflammation in hypertension: targeting lipid mediators unbalance and nitrosative stress. Curr. Hypertens. Rev..

[35] Walsh G.M., Leane D., Moran N., Keyes T.E., Forster R.J., Kenny D. (2007). S-Nitrosylation of platelet alphaIIbbeta3 as revealed by Raman spectroscopy. Biochemistry.

[36] Nilsson S., Kaniowska D., Brakebusch C., Fassler R., Johansson S. (2006). Threonine 788 in integrin subunit beta1 regulates integrin activation. Exp. Cell Res..

[37] Wojdyla K., Wrzesinski K., Williamson J., Fey S.J., Rogowska-Wrzesinska A. (2016). Acetaminophen-induced S-nitrosylation and S-sulfenylation signalling in 3D cultured hepatocarcinoma cell spheroids. Toxicol. Res. (Camb).

[38] Bottcher R.T., Stremmel C., Meves A., Meyer H., Widmaier M., Tseng H.Y. (2012). Sorting nexin 17 prevents lysosomal degradation of beta1 integrins by binding to the beta1-integrin tail. Nat. Cell Biol..

[39] McCall-Culbreath K.D., Li Z., Zutter M.M. (2008). Crosstalk between the alpha2beta1 integrin and c-met/HGF-R regulates innate immunity. Blood.

[40] Yao Q., Liu J., Zhang Z., Li F., Zhang C., Lai B. (2018). Peroxisome proliferator-activated receptor gamma (PPARgamma) induces the gene expression of integrin alphaVbeta5 to promote macrophage M2 polarization. J. Biol. Chem..

[41] Imhof B.A., Aurrand-Lions M. (2004). Adhesion mechanisms regulating the migration of monocytes. Nat. Rev. Immunol..

[42] Huang L.H., Melton E.M., Li H., Sohn P., Jung D., Tsai C.Y. (2018). Myeloid-specific Acat1 ablation attenuates inflammatory responses in macrophages, improves insulin sensitivity, and suppresses diet-induced obesity. Am. J. Physiol. Endocrinol. Metab..

[43] Ariano C., Riganti C., Cora D., Valdembri D., Mana G., Astanina E. (2022). TFEB controls integrin-mediated endothelial cell adhesion by the regulation of cholesterol metabolism. Angiogenesis.

[44] Zhang Z., Xie X., Yao Q., Liu J., Tian Y., Yang C. (2019). PPARdelta agonist prevents endothelial dysfunction via induction of dihydrofolate reductase gene and activation of tetrahydrobiopterin salvage pathway. Br. J. Pharmacol..

[45] Jackson S.K., Thomas M.P., Smith S., Madhani M., Rogers S.C., James P.E. (2004). *In vivo* EPR spectroscopy: biomedical and potential diagnostic applications. Faraday Discuss..

[46] Huang B., Chen C. (2010). Detection of protein S-nitrosation using irreversible biotinylation procedures (IBP). Free Radic. Biol. Med..

