# Supplementary material for: Free fatty acids stabilize integrin β1via S-nitrosylation to promote monocyte–endothelial adhesion
Source: J Biol Chem. 2022 Dec 5;299(1):102765. doi: 10.1016/j.jbc.2022.102765 (PMC9808002; doi:10.1016/j.jbc.2022.102765)
Supplement: Supplemental Tables S1–S2 and Figure S1 [file mmc1.doc]

**Supporting Information**

| **Name** | **Sense** | **Anti-sense** |
| --- | --- | --- |
| **iNOS A** | GCACAUCUGCAGACACAUACUTT | AGUAUGUGUCUGCAGAUGTGCTT |
| **iNOS B** | GCUGAAAUCCCAGCAGAAUCUTT | AGAUUCUGCUGGGAUUUCAGCTT |
| **integrin β_1_** | GCACGAUGUGAUGAUUUAGAATT | UUCUAAAUCAUCACAUCGUGCTT |
| **c-Cbl** | CGTTTGGGTCAGTGGGCTATT | TAGCCCACTGACCCAAACGTT |
| **Scrambled** | UUCUCCGAACGUGUCACGUTT | ACGUGACACGUUCGGAGAATT |

**Table 1: list of siRNA sequences**

| **Name** | **Forward primer (5’-3’)** | **Reverse primer (5’-3’)** |
| --- | --- | --- |
| **iNOS** | GCAGCTGGGCTGTACAAA | AGCGTTTCGGGATCTGGAAT |
| **integrin α_4_** | GGTCCCAGGCTACATCGTTT | GGGGTAAGGATGTCTCGCAC |
| **integrin β_1_** | ATGCCAAATCTTGCGGAGAA | CATCGTGCAGAAGTAGGCATT |
| **GAPDH** | ACCACAGTCCATGCCATCAC | TCCACCACCCTGTTGCTGTA |

**Table 2: list of primers used in RT-qPCR experiment**


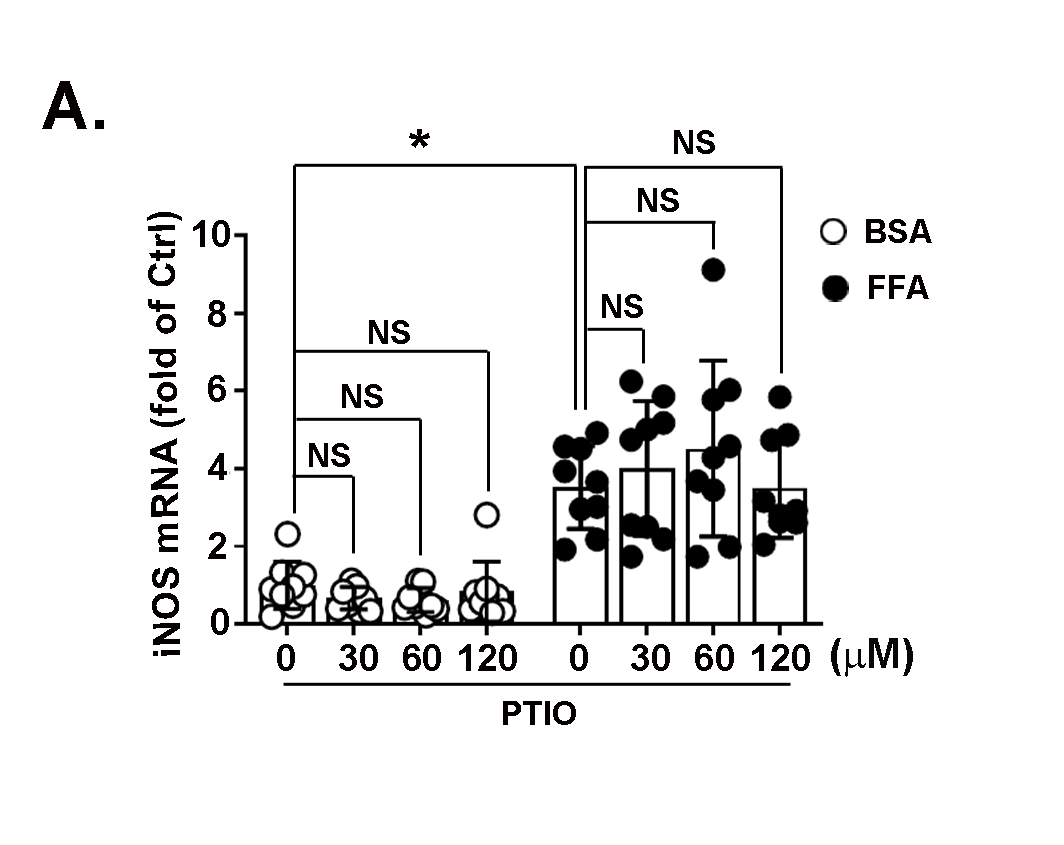


**Figure S1: Effect of PTIO on iNOS mRNA.** Raw264.7 cells were pretreated with the indicated concentrations of PTIO for 1h, then exposed to FFA (1 mM) for 24h. The cell lysates were analyzed with RT-qPCR. *, p<0.05, NS: not significant.
